# Using Data Augmentation to Improve the Generalization Capability of an Object Detector on Remote-Sensed Insect Trap Images

**DOI:** 10.3390/s24144502

**Published:** 2024-07-11

**Authors:** Jozsef Suto

**Affiliations:** 1 Department of Informatics Systems and Networks, Faculty of Informatics, University of Debrecen, Kassai Street, 26, 4028 Debrecen, Hungary; suto.jozsef@inf.unideb.hu; Tel.: +36-52-512-900 (ext. 75016); 2 Department of IT, Eszterhazy Karoly Catholic University, Leanyka Street, 4, 3300 Eger, Hungary

**Keywords:** automated trap, insect counting, data augmentation, YOLOv5

## Abstract

Traditionally, monitoring insect populations involved the use of externally placed sticky paper traps, which were periodically inspected by a human operator. To automate this process, a specialized sensing device and an accurate model for detecting and counting insect pests are essential. Despite considerable progress in insect pest detector models, their practical application is hindered by the shortage of insect trap images. To attenuate the “lack of data” issue, the literature proposes data augmentation. However, our knowledge about data augmentation is still quite limited, especially in the field of insect pest detection. The aim of this experimental study was to investigate the effect of several widely used augmentation techniques and their combinations on remote-sensed trap images with the YOLOv5 (small) object detector model. This study was carried out systematically on two different datasets starting from the single geometric and photometric transformation toward their combinations. Our results show that the model’s mean average precision value (mAP50) could be increased from 0.844 to 0.992 and from 0.421 to 0.727 on the two datasets using the appropriate augmentation methods combination. In addition, this study also points out that the integration of photometric image transformations into the mosaic augmentation can be more efficient than the native combination of augmentation techniques because this approach further improved the model’s mAP50 values to 0.999 and 0.756 on the two test sets, respectively.

## 1. Introduction

In the realm of agricultural pest management, defending crops from insect pests is a continuous challenge. The impact of insect pests on crop yields is substantial, leading farmers to employ insecticides at predetermined intervals, irrespective of the actual pest population size [1,2]. Spraying serves as the primary control method against most insect types. For example, the authors of [3] stated that 70 percent of the applied spraying was directed at codling moths in apple orchards. A more efficient approach than periodic spraying would be to only use insecticides at times when the pest population rises above a predefined threshold. This requires an accurate forecast of the pest population and brings both environmental benefits (reduced insecticide use) and economic advantages (cost savings, etc.). To obtain quantitative data for predicting pest density, different trap types (pheromone-based, light traps, etc.) can be employed [4,5]. In pheromone-based traps, a pheromone capsule attracts male insects to the trap, where they become stuck on sticky paper. *Cydia pomonella* (codling moth) is the most harmful pest of apple and pear in Hungary and in other countries [6,7]. Therefore, we collected and analyzed trap images of this pest, also focusing on the type of trap image.

Manual monitoring of these sticky papers conducted by experts who count trapped insects has well-documented drawbacks, including the need for skilled personnel, time consumption, and high costs [8,9]. Moreover, manual counting lacks continuous feedback, resulting in low temporal resolution for insect pest population tracking. Recognizing these limitations, researchers and industrial members turned toward smart solutions, leading to the development of embedded system-based automated traps supported by machine learning [10,11].

Apart from image capturing devices, a high-precision insect counting method is also crucial. Viewing insect counting as a special case of object detection, researchers have found that the widely used one- and two-stage-deep object detectors can be effectively used for this task [9,12,13]. Among these, the You Only Look Once (YOLO) single-stage object detector model has been applied many times. Li et al. [14] recommended YOLOv5 based on its high accuracy (above 99%) on the images of the Baidu AI insect detection database [15]. Reference [16] compared various deep object detectors, including a Faster Region-based Convolutional Neural Network (R-CNN) and Single-Shot Multibox Detector (SSD). Out of those models, the Faster R-CNN achieved the highest mean average precision (mAP) but with longer decision times, while the SSD was the fastest, with a lower mAP. mAP is a widely accepted performance metric for object detectors. This metric comes from the average precision (AP) across all classes in the test set where AP is determined by the area under the precision–recall curve [10].

Despite the results in earlier studies being encouraging, many studies suffer from the lack of trap images, which is a general issue in the field of insect detection and counting [17,18]. To address this problem, data augmentation is an obvious option. Over time, numerous data augmentation approaches have been introduced. Many of them have also been employed in studies related to pest detection. For example, researchers such as Albanese et al. [19] and Kasinathan and Uyyala [20] utilized image transformation techniques (e.g., image translation, flipping) on training images. Even though the idea of artificial data enrichment has appeared in more articles dealing with insect pest detection and counting, relatively little is known about how the possible combinations of augmentation techniques affect the performance of object detectors [9]. Therefore, we conducted an experimental study including six widely used data augmentation techniques to examine how the methods and their combinations modify the detector model’s performance. 

In a significant portion of practical applications, the model is embedded into the remote sensing device. It is well known that the computational capacity of such edge devices is limited, making the usage of complex object detector models non-optimal. Moreover, many edge devices are not even capable of running more complex object detector models. Therefore, as test model, the popular YOLO version 5 (YOLOv5) object detector was employed, trained on a limited set of own trap images and tested on two different sets of trap images. Starting from the individual augmentation methods through their different combinations, we arrived at a data enrichment approach that integrates photometric image transformation methods into the mosaic augmentation. The contributions of this study are the following:

- Our findings indicate that by using the appropriate combination of augmentation methods, YOLOv5’s mean average precision value (mAP50) could be further increased from 0.844 to 0.992 and from 0.421 to 0.727 on the two remote-sensed trap image datasets.
- This study reveals that incorporating photometric image transformations into the mosaic augmentation can be more effective than the standard combination of augmentation techniques.
- The experimental results show that this approach surpasses the native combination of augmentation techniques and YOLOv5’s built-in image enrichment process with HSV (hue, saturation, and value) and mosaic augmentations in efficiency.

## 2. Materials and Methods

### 2.1. The YOLO Model Family

Insect counting belongs into the object detection research field, which is a highlighted area in computer vision. Object detection typically involves identification of visual object categories such as faces, humans, or vehicles. In the last decade, various detector algorithms have been proposed for this task. These methods belong to different classes: computer vision-based [21], single-stage object detectors like the models of YOLO [22], and two-stage object detectors like Fast and Faster R-CNN [23]. The evolution of CNN-based object detection started with the R-CNN in 2014. It was followed by several single- and multi-stage deep object detector models. Currently, the latest members of the YOLO family are considered state-of-the-art models due to their fast decision time and accurate object localization ability. Therefore, we employed the YOLOv5 as object detector in this study.

YOLOv5 is an improved version of the predecessor YOLOv3 developed by Glen Jocker (Ultralytics) in 2020. The fifth version of YOLO includes various model architectures (YOLOv5s (small), YOLOv5m (medium), YOLOv5l (large), and YOLOv5x (extra-large)) that differ mainly in the number of convolutional layers. All members of the YOLOv5 family are trained on the COCO dataset (https://cocodataset.org/#home, accessed on 4 March 2024). Considering that pest counting occurs in the edge device in many monitoring systems, factors such as inference time (critical for battery life) and computational resource requirements (e.g., volatile memory) become crucial. Taking these facts into consideration, we selected the small version (YOLOv5s) as reference model.

The model was trained using minibatch-based stochastic gradient descent (SGD). The mini-batch size was adjusted to the GPU memory in the used computer, which was 16. The SGD parameters included a momentum of 0.9 and a weight decay of 0.0001, with an initial learning rate set at 0.01. The training process incorporated a stopping criterion of “no improvement in 20 epochs”. Although the epoch limit was 300, the training process has not reached this limit in most test cases. 

### 2.2. Data Augmentation

In machine learning, it is widely recognized that enhancing the quantity of data contributes significantly to improving the generalization performance of a model. Data augmentation is particularly important in situations where the number of samples in the training set is limited since deep models are rather “data hungry”, requiring a substantial volume of information for fine-tuning weights. Consequently, the process of data augmentation (also known as artificial data enrichment) holds a pivotal role in the learning process by extending the available training dataset. 

Due to the diverse sticky paper (and image) regions where moths can be captured, achieving translation invariance is crucial for an insect counter model. Additionally, to manage size discrepancies, the model must be scaling invariant. Therefore, geometric augmentation methods play a vital role in enhancing the generalization capability of the detector model. In addition to geometric augmentation methods, photometric augmentation techniques, such as noise pollution and adjustment of brightness and contrast, aid in addressing texture differences among captured insects. Although photometric augmentation helps in better handling texture differences among the same and different pest species, our experience shows that the difficulty arises from the fact that insects of the same type adhere to the sticky sheet in different poses, resulting in their textures not being completely identical.

Object detectors created in recent years already incorporate geometric (random scaling, translation, flipping, and rotation) and photometric augmentation techniques (e.g., brightness, contrast, saturation adjustment). Moreover, modern object detectors consider additional augmentation methods like random erase, mixup, or mosaic augmentation. From the standpoint of insect detection, mosaic augmentation can help handling the “small object detection problem”. This approach involves generating a new image by combining specific ratios of four other images [24,25]. The newly created image displays many more objects at a reduced size. This creates more complex and diverse training examples, which can improve the model’s performance for small-sized objects.

In our experimental investigations, the translation, rotation, and mosaic augmentations were used out of the geometric methods. The number of horizontal and vertical translations (Δx,Δy) is randomly selected from a uniform distribution Δx,Δy∈U(−0.2,0.2) where the maximum translation can be 0.2 times the original image size. The degree of image rotation is also randomly selected, θ∈U(−90,90). In the case of the mosaic image generation, the four images are randomly selected where the crop offset could be up to 30% of the “merged” image size. 

Images captured by traps in the field are affected by diverse illumination conditions due to variations in daylight, weather conditions, and landscape elements causing shadows [26]. To mitigate the illumination differences between images, brightness–contrast adjustment and gamma correction [27] are employed. Gamma correction transforms each pixel of an image as described by (1), where f(x,y) represents the scaled input pixel (between 0 and 1), and c is the gain. In our experiment, c was the constant one without change, while gamma comes from the following uniform distribution: γ∈U(0.2,3.0). Depending on γ, the transformation converts the original pixel intensities into different output ranges.

(1) $$f^{*}(x,y)={cf(x,y)}^{\gamma }$$

Brightness and contrast adjustments are commonly used image processing techniques that can be performed in the same step (2). In the formula, α and β are called gain and bias, respectively. The former is responsible for contrast while the latter adjusts the brightness of the output image. In our experimental analysis, both parameters were randomly selected: α∈U(0.5,2.0) and β∈U(−50,50).

(2) $$g\left(x,y\right)=\alpha f\left(x,y\right)+\beta$$

**Figure 1:**
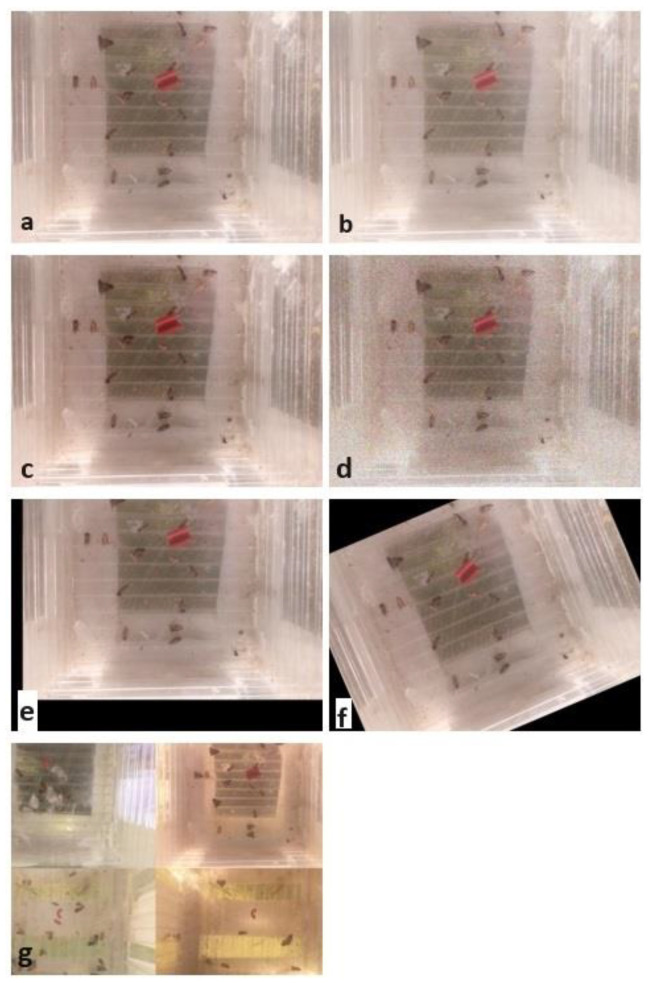
Augmented sticky trap image (**a**) with gamma adjustment (**b**), brightness and contrast adjustment (**c**), noise pollution (**d**), translation (**e**), rotation (**f**), and mosaic (**g**) techniques.

The automated trap-based image capturing is also affected by noise due to oscillations and impurities on the camera. If only a small training set is available, deep models cannot handle noisy test samples well in real applications [28]. Moreover, models that are trained on high-quality images often produce inferior results, as they struggle to handle the inherent variability. In this case, an opportunity for improvement is to train the model by adding some type of random noise to the training dataset [29]. Adding noise to the images means that the model is less able to memorize training samples because they slightly change randomly. The three widely used noise types are the Gaussian (or white noise), Speckle, and Salt and Pepper noise. In this work, we used Gaussian noise as one of the image augmentation techniques. Here, a significant question is the noise amount, taking into consideration that too small an amount of noise has no effect, whereas too much noise suppresses the useful information in the image. We used a Gaussian distribution for noise generation with mean 1 and standard deviation of 0.2. An illustration of the data augmentations used in this study can be seen in Figure 1.

### 2.3. Datasets

In remote sensing applications, image quality can vary significantly depending on factors such as the type of image capture device used and environmental lighting conditions. These factors are significant, and they need to be taken into consideration at the time of model construction because image quality discrepancy influences the recognition accuracy. This is especially true for the data available to us because there is a significant quality difference between the images in the two test datasets due to the different conditions of image-taking and differences between remote sensing devices.

**Figure 2:**
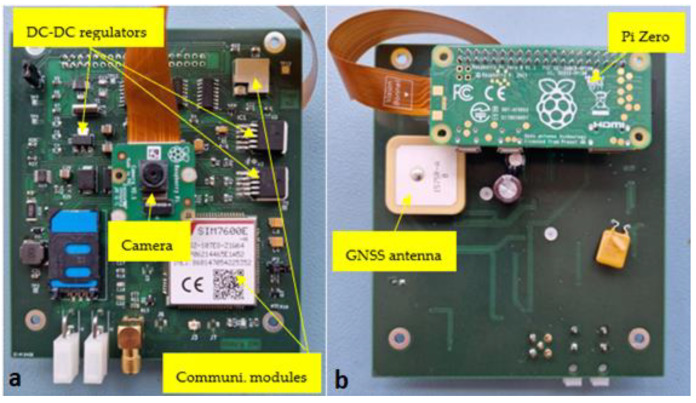
Our remote sensing unit used for image acquisition: (**a**) front and (**b**) back sides.

The training dataset used in this study consists of 330 sticky paper images acquired by our own special sensing device dedicated to remote insect pest monitoring [30]. It comprises a Raspberry Pi Zero W, a special plug-in board dedicated to automated sticky traps, and a Raspberry Pi Camera v2. A representative figure of the device is depicted in Figure 2. The images in one out of the two test sets were also acquired by the same device. It contains 150 sample images. 

**Figure 3:**
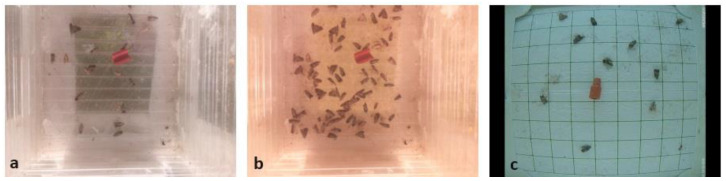
Sample images from the training set (**a**), first test set (**b**), and second test set (**c**).

In order to present a general picture about the effectiveness of data augmentation, a second test set was also included in the investigation. It comes from the Roboflow’s public repository and incorporates 233 remote-sensed stick paper images with the annotation files (available at: https://github.com/suto-jozsef/trap_images.git, accessed on 8 May 2024). Visually, the images in this set differ from the elements of the training set, but even so, those images were the most similar to our remote-sensed trap images among the publicly available images (Figure 3). Since the image capture conditions and devices were different in the case of the two test sets, the possible positive effect of image augmentation was even more noticeable. 

### 2.4. Evaluation Metrics

Inside the insect pest detection and counting research field, a critical question arises: how can the efficiency of the insect detector model be measured? In 2016, automated insect detection and counting was a quite rudimentary research field, lacking a standardized protocol for evaluating insect counting algorithms [31]. Consequently, researchers turned to metrics from other areas of computer vision, such as pedestrian detection. One widely accepted performance metric for object detectors in computer vision is the mean average precision (mAP), calculated as the average precision (AP) metric of each class, where the average precision comes from the area under the curve of precision (3) and recall (4) with threshold value k.

(3) $$p(k)=\frac{\#\;true\;positive\;detections}{total\;number\;of\;detections}$$

(4) $$r(k)=\frac{\#\;true\;positive\;detections}{total\;number\;of\;ground\;truth\;boxes}$$

For precision–recall curve generation, we must have information on the number of true and false detections. The determination of true and false detections is based on the intersection-over-union (IoU), which is a metric of the overlapping areas between the ground truth box and the predicted box. In most studies, a proposed bounding box is considered a true positive if the IoU value is equal to or higher than 0.5. Otherwise, it is classified as a false positive [16,32]. In this paper, we utilized two versions of mAP to assess the performance changes in the YOLO model. The first version uses the popular 0.5 IoU threshold value (mAP@50) while the second version calculates the average AP from 0.5 IoU to 0.95 IoU with a step size of 0.05 (mAP@50-95). 

## 3. Results and Discussion

**Figure 4:**
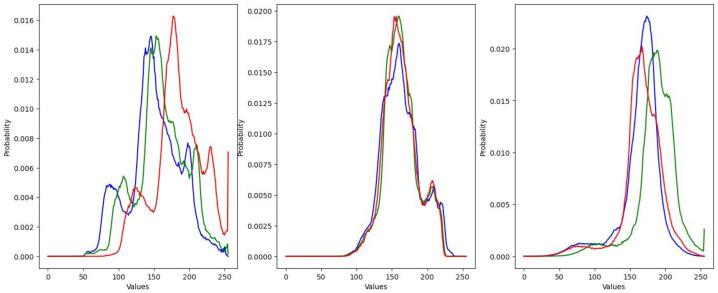
Color histogram the test images from the training set (**left**), first test set (**center**), and second test set (**right**). The red, green, and blue colors refer to the RGB color spaces.

The investigation started with the determination of initial mAP values that came from the performance of the YOLOv5s on the test sets with the original without data augmentation. Thereafter, the gamma correction, brightness and contrast adjustment, noise pollution, translation, rotation, and mosaic augmentation methods were examined one-by-one with gradually increasing data enrichment (multiple of the original training data size). As mentioned in Section 2, there is a strong randomness in the augmentation process. In order for experiments to be repeatable, the same random seed was used. Gamma correction and brightness–contrast adjustment can be applied channel-wise on images and can even be used in different ways on bit planes of a multi-channel image. After the examination of the red, green, and blue color histograms of images in the training and test sets, it was well visible that they had similar color distribution in each channel (Figure 4). Therefore, the same mapping function was used for all of them. 

**Figure 5:**
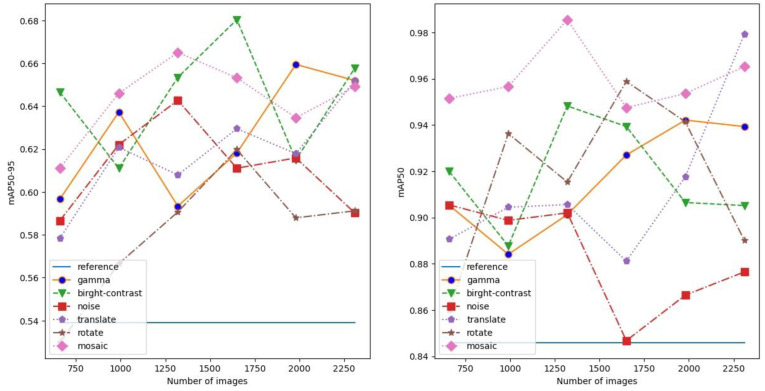
The mAP50-95 and mAP50 values of the model on the first test set.

**Figure 6:**
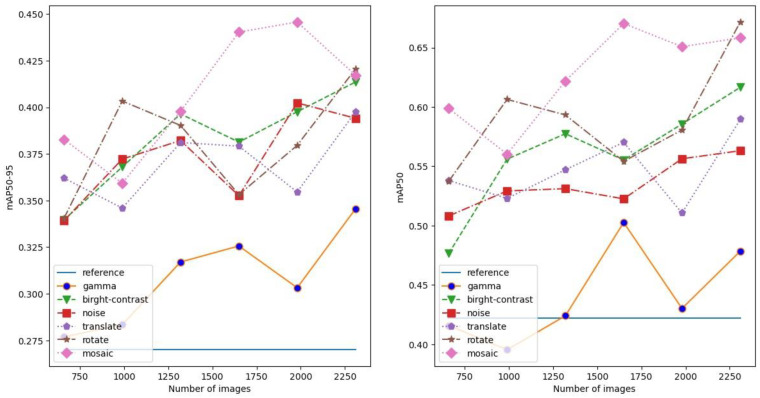
The mAP50-95 and mAP50 values of the model on the second test set.

The experimental results with the individual augmentation methods and without augmentation can be seen in Figure 5 and Figure 6. In all cases, when augmentation is used, the original training set is also incorporated into the augmented training set. The former figure shows the performance of the model on the first test set while the latter is the result on the second test set. Both the mAP50 and mAP50-95 metrics are visualized to understand better the effects of augmentation. In both figures, the straight line indicates the mAP50 and mA50-95 values of the model without augmentation. During the experiments, the training dataset size was increased continuously. At first, the original training set size was duplicated using the augmentation methods. Since the size of the original training set was small, we decided to increase it by a multiple in each step. In every case, the model was initialized in the same way, and the highest test result was recorded. 

Due to the randomness in the augmentation techniques, the graphs are rather wavy. However, by carefully observing the figures, some interesting conclusions can be drawn. On the first test set, the model achieved significantly higher mAP values than on the second set. This can be explained by the higher similarity between the images in the training set and the elements of the first test set. In addition, the second set contains more images with lower image quality.

On the first test set all image augmentation techniques increased the model’s mAP50-95 values. In the case of the second set, a similar trend can be observed but the gamma correction was not as efficient as in the first case. Comparing the graphs, we can also see that the mosaic augmentation stands out among the augmentation techniques. 

The degree of data enrichment is another important question. In the case of the first test set, the increasing data enrichment did not bring a clear improvement while a slightly improving mAP trend could be observed in the case of the second set. 

Continuing the investigation, we tested the augmentation methods in pairs. A total of 330 images were generated, with all augmentation techniques and their combinations plus the original training set used to train YOLOv5. Since here the size of the training set did not change (only its composition), the achieved metrics are presented in Table 1 and Table 2. In the case of the first test set, each augmentation method pair was examined, while in the case of the second set, the gamma correction was omitted from the investigation due to the earlier finding. If we compare the content of Table 1 with the results in Figure 5, we see that only the mosaic–noise pair and the combination of the mosaic and brightness–contrast adjustment techniques brought considerable efficiency growth.

If we examine Table 2, we clearly see that the noise and mosaic augmentation pair was the most efficient, bringing substantial improvement in both metrics. Besides this pair, the brightness–contrast plus mosaic pair and the translation plus mosaic pair seem a successful pairing (considering the number of images). The results also show that the mosaic augmentation method is the most efficient out of the tested methods. Since the mosaic and noise pollution-based augmentation pair proved effective on both test datasets, we continued the investigation with this pair. 

In the next phase, the mosaic–noise pair is extended with an additional augmentation technique. Therefore, the current training set consists of the original 330 training images plus the augmented images generated by the methods. The results of this approach can be found in Table 3 and Table 4. This approach already had a different effect on the two test datasets. Merging the three augmentation methods did not prove effective on the first set. However, it further increased the mAP50 and mAP50-95 metrics on the second test set where the combination of the mosaic, noise, and brightness–contrast adjustment was the most efficient. It may seem obvious to add one more augmentation method to this trio, but our further investigation showed that this causes performance degradation. We believe that the reason for this is the significant shift in the proportion of images in the training dataset towards generated images compared to the original dataset. In the augmented set, the generated images constitute multiple times the number in the original image set. Since noise is introduced during augmentation, excessive data augmentation can lead to model overfitting instead of improving the model’s generalization ability.

The implementation of the YOLOv5 training process also includes different kinds of augmentation techniques that can be optionally used and parameterized. In order to obtain a clear picture of the effectiveness of the augmentation method combinations presented above, the model was tested with the “built-in” image augmentation process incorporating the HSV and mosaic techniques. The model supported by the built-in augmentation achieved 0.973 mAP50 and 0.678 mAP50-95 on the first test set and 0.659 mAP50 and 0.454 mAP50-95 on the second test set, respectively. The comparison of the reference values with the content of Table 1 to 4 points out that the model’s performance can be further increased if we use additional carefully selected augmentation methods, not only the YOLO’s built-in mechanism. 

### Modified Mosaic Augmentation

**Figure 7:**
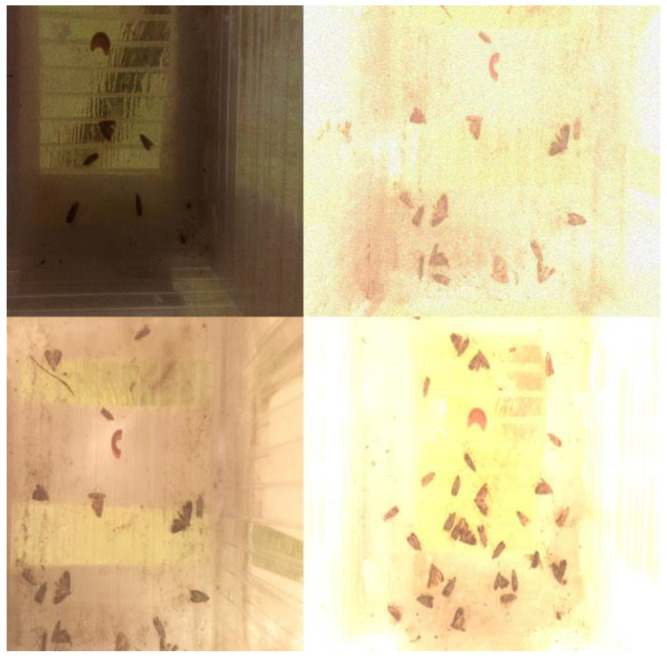
A sample mosaic image where brightness–contrast adjustment and noise pollution are randomly applied on the sub-images.

Since the mosaic augmentation technique was the most efficient out of the tested methods, we introduced an additional augmentation strategy (modified mosaic) where the elements of the mosaic image were randomly modified by the earlier-used brightness–contrast adjustment and noise pollution (Figure 7). The probabilities of using brightness–contrast adjustment and noise pollution were 0.8 and 0.3. 

**Figure 8:**
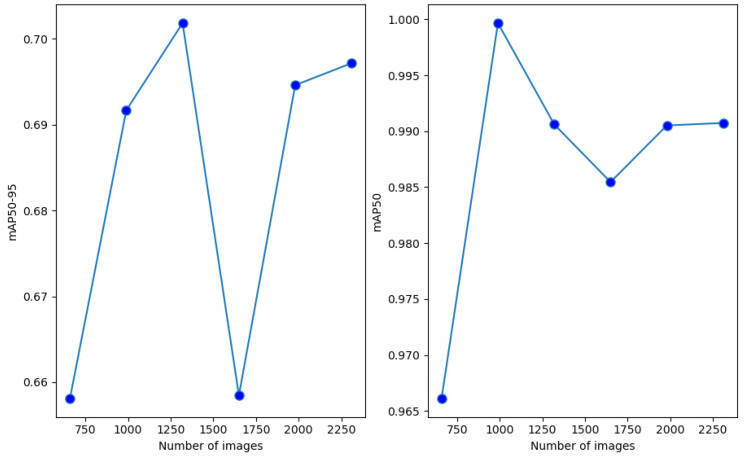
mAP50-95 and mAP50 values achieved with the modified mosaic augmentation on the first test set.

**Figure 9:**
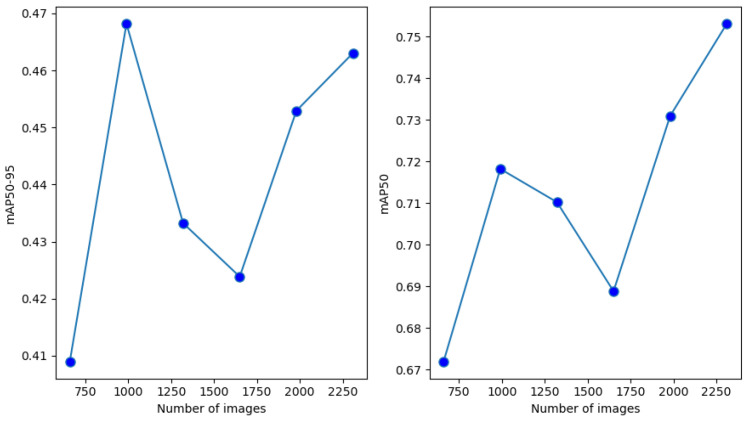
mAP50-95 and mAP50 values achieved with the modified mosaic augmentation on the second test set.

In the last experiment, we used only those specific mosaic images to extend the original training set. Similarly, as in the case of the single-augmentation methods, the original training set size was increased by a multiple in each step. The mAP50 and mAP50-95 values achieved on the two test sets can be seen in Figure 8 and Figure 9.

The figures show that the modified mosaic augmentation brought higher mAP50 and mAP50-95 than any single augmentation technique. Similarly to Figure 5 and Figure 6, in the case of the first test set, the increasing data enrichment did not bring a clear improvement while a slightly improving trend can be observed in the case of the second test set. Finally, it also can be observed that the modified mosaic augmentation can be even more efficient than merging different augmentation techniques (see Table 3 and Table 4). 

The effectiveness of the modified mosaic augmentation lies in integrating the advantages of geometric and texture augmentations into the original mosaic augmentation. This creates even more complex and diverse training examples compared to the original method, further enhancing the model’s generalization ability. 

## 4. Conclusions

In this paper, we applied gamma adjustment, brightness–contrast adjustment, noise pollution, translation, rotation, and mosaic image augmentation methods and their combinations to manually increase the original training data size. To investigate the efficiency of different augmentation approaches, we used the YOLOv5 small object detector. To measure the effectiveness of the YOLOv5 model, the mAP50 and mAP50-95 metrics were used. The experimental results from two test sets that consisted of remote-sensed trap images presented how the individual augmentation methods are capable of increasing the mAP of the model relative to its performance without augmentation. The results also demonstrated that the YOLOv5 model’s performance can be further increased if additional carefully selected augmentation methods, and not only the built-in mechanism, are used. Finally, we proposed the integration of photometric image transformations into the mosaic augmentation and our experimental results show that this approach can be even more efficient than the native combination of augmentation techniques.

## Figures and Tables

**Table 1:** Measurement results with augmentation method pairs on the first test set.

Augmentation Strategy	mAP50	mAP50-95	Number of Images
Gamma + brightness–contrast	0.922	0.605	990
Gamma + noise	0.914	0.609	
Gamma + translation	0.878	0.591	
Gamma + rotation	0.897	0.567	
Gamma + mosaic	0.981	0.679	
Brightness–contrast + noise	0.920	0.599	
Brightness–contrast + translation	0.924	0.615	
Brightness–contrast + rotation	0.919	0.595	
Brightness–contrast + mosaic	0.989	0.683	
Noise + translation	0.928	0.605	
Noise + rotation	0.949	0.616	
Noise + mosaic	0.992	0.672	
Translation + rotation	0.968	0.639	
Translation + mosaic	0.985	0.668	
Rotation + mosaic	0.980	0.675	

**Table 2:** Measurement results with augmentation method pairs on the second test set.

Augmentation Strategy	mAP50	mAP50-95	Number of Images
Brightness–contrast + noise	0.514	0.349	990
Brightness–contrast + translation	0.471	0.335	
Brightness–contrast + rotation	0.451	0.323	
Brightness–contrast + mosaic	0.695	0.459	
Noise + translation	0.606	0.409	
Noise + rotation	0.549	0.362	
Noise + mosaic	0.701	0.481	
Translation + rotation	0.540	0.362	
Translation + mosaic	0.647	0.447	
Rotation + mosaic	0.594	0.383	

**Table 3:** Measurement results with three augmentation methods on the first test set.

Augmentation Strategy	mAP50	mAP50-95	Number of Images
Mosaic + noise + gamma	0.981	0.647	1320
Mosaic + noise + brightness–contrast	0.988	0.686	
Mosaic + noise + translation	0.985	0.654	
Mosaic + noise + rotation	0.979	0.678	

**Table 4:** Measurement results with three augmentation methods on the second test set.

Augmentation Strategy	mAP50	mAP50-95	Number of Images
Mosaic + noise + brightness–contrast	0.727	0.466	1320
Mosaic + noise + translation	0.709	0.460	
Mosaic + noise + rotation	0.714	0.464	

## Data Availability

The training and the first test datasets analyzed during the current study are not publicly available due to the restriction of the data owner.
